# Generation of Sequencing Libraries for Structural Analysis of Bacterial 5′ UTRs

**DOI:** 10.1016/j.xpro.2020.100046

**Published:** 2020-06-06

**Authors:** Dmitriy Ignatov, Karolis Vaitkevicius, Jörgen Johansson

**Affiliations:** 1 Department of Molecular Biology, Umeå University, 901 87 Umeå, Sweden; 2 Umeå Centre for Microbial Research, Umeå University, Umeå, Sweden; 3 Laboratory for Molecular Infection Medicine Sweden (MIMS), Umeå University, Umeå, Sweden

## Abstract

The structure of 5′ untranslated regions (5′ UTRs) of bacterial mRNAs often determines the fate of the transcripts. Using a dimethyl sulfate mutational profiling with sequencing (DMS-MaPseq) approach, we developed a protocol to generate sequence libraries to determine the base-pairing status of adenines and cytosines in the 5′ UTRs of bacterial mRNAs. Our method increases the sequencing depth of the 5′ UTRs and allows detection of changes in their structures by sequencing libraries of moderate sizes.

For complete details on the use and execution of this protocol, please refer to Ignatov et al. (2020).

## Highlights

• A DMS-MaPseq-based protocol to analyze the structures of 5′ UTRs of bacterial mRNAs
• Fast assembly of sequence libraries for RNA structural determination
• Protocol can be used for eukaryotic cells after minor modification

## Graphical Abstract

**Figure:**
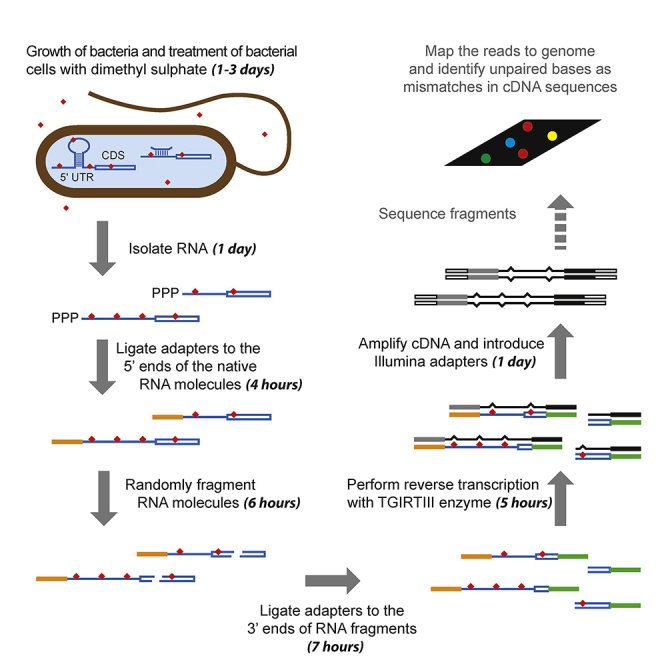

## Teaser

The protocol by Ignatov et al, shows how to use DMS as a mutational probe and how to generate sequence libraries to determine the structures of bacterial 5′-untranslated regions.

## BEFORE YOU BEGIN

A number of approaches, collectively known as Structure-seq, combine chemical probing of RNA structure with high-throughput sequencing. These methods allow study of RNA structures inside living organisms at the whole-transcriptome level to characterize their “RNA structurome” (Bevilacqua et al., 2016). DMS (dimethyl sulphate) reagent can be used to probe RNA structures and protein:RNA interactions inside living cells (Wells et al., 2000). DMS selectively methylates N1 of adenine and N3 of cytosine if these nucleotides are not base-pairing or interacting with proteins (Inoue and Cech, 1985, Lempereur et al., 1985). The modified bases can be detected by incorporation of non-complementary nucleotides during cDNA synthesis. These approaches, termed MaP for ʹMutational profilingʹ, calculate DMS reactivity rates for each nucleotide as the percentage of mutations in the cDNA library (Smola et al., 2015). After normalization, the DMS reactivity rates can serve as a measure of the base-pairing status of individual nucleotides (Homan et al., 2014, Siegfried et al., 2014, Zubradt et al., 2017). In this protocol, we described details of using DMS-MaPseq to generate sequence libraries for global structural analysis of bacterial mRNAs.

### Gel Purification of 5′ RNA Adapter (3′RA)

**Timing: 8 h**

***Note:*** The 5′RA is an RNA adapter. To prevent self-ligation, it has 3′-OH and 5′-OH ends. However, due to incomplete synthesis or degradation, truncated variants can be observed. These truncated versions are prone to self-ligation and formation of long RNA molecules that can compromise the size selection step of the library preparation. Therefore, it is recommended to purify the 5′RA on a denaturing polyacrylamide (PAA) gel.

1. Pour 15% Urea-PAA gel using plates and 5 well 0.75 mm wide combs of the Mini Protean system (Biorad):

**Table:** 

Reagent	Amount
15% Urea-PAA stock	9.91 mL
10 % Ammonium persulfate	80 μL
TEMED	10 μL
**Total**	**10 mL**

2. Mix 10 μL of 100 μM 5′RA with 10 μL 2× RNA loading dye (ThermoFisher) and apply to the gel. Run the gel until the bromophenol blue runs the 2/3 of the gel. Stain for 10 min with SYBR Gold (ThermoFisher) and visualize on a blue light transilluminator. Cut the gel fragments with the band corresponding to the major fragment.
3. Disintegrate the gel fragments: with a hot 0.6 × 25 mm needle (BD), punch a hole in the bottom of a 0.5 mL tube. Put the gel fragments to this 0.5 mL tube and insert it into a 2 mL tube. Centrifuge for 10 min at > 10 000 g. During the centrifugation the gel crushes by passing through the small hole.

***Alternatives:*** Gel breaking can also be achieved using commercially available Gel Breaker Tubes (IST Engineering).

4. Add 300 μL of 300 mM NaCl solution to the crushed gel in the 2 mL tube and incubate for 2 h at 50°C and shaking at 300 rpm.
5. Transfer the gel slurry to the Costar Spin-X column (Merck) and centrifuge for 3 min at 10,000 × *g* to remove the gel pieces.
6. Precipitate RNA by adding 2 μl GlycoBlue coprecipitant (ThermoFisher) and 900 μL 100% EtOH, incubating at 20°C for 1 h and spinning at 20 000 g for 30 min at 4°C.
7. Resuspend the purified RNA adaptor in 20 μL RNase-free water, measure its concentration on Nanodrop spectrophotometer (ThermoFisher) and adjust the concentration to 15 μM.

***Note:*** For the 1 nmol input of RNA adaptor expect to recover 0.4 to 0.6 nmol of the purified RNA adaptor (25–40 μL of 15 μM purified RNA adaptor).

### 5′-Adenylation of 3′ DNA Adapter (3′DA)

**Timing: 3 h**

8. Set up the following reaction in a 0.5 mL tube to adenylate the 3′ DA using the 5′ DNA Adenylation Kit (NEB):

**Table:** 

Reagent	Amount, μL
3′DA (100 μM)	2
10× 5′ DNA Adenylation Reaction Buffer	4
1 mM ATP	4
Mth RNA Ligase	4
Nuclease-free Water	26
**Total**	**40**

9. Incubate in a thermocycler with the heated lid at 65°C for 1 h.
10. Inactivate the enzyme by incubation at 85°C for 5 min.
11. Purify the adenylated 3′DA using Oligo Clean & Concentrator kit (Zymo).
12. Measure the oligonucleotide concentration on Nanodrop spectrophotometer (ThermoFisher), adjust the concentration to 15 μM.

## Key Resources Table

**Table:** 

REAGENT or RESOURCE	SOURCE	IDENTIFIER
**Chemicals, Peptides, and Recombinant Proteins**		

Brain Heart Infusion (BHI) Broth	BD	237500
Tris-Borate-EDTA buffer	Merck	T4415-1L
MOPS	Merck	69947-500G
MES monohydrate	Merck	69889-250G
EDTA (0.5 M), pH 8.0	ThermoFisher	AM9262
Dimethyl sulphate	Merck	41610-100ML
Isoamyl alcohol	Scharlau	AL0285
2-Mercaptoethanol	Merck	M3148-500ML
Acid phenol/Chloroform (pH 4.5)	ThermoFisher	AM9722
SYBR™ Gold Nucleic Acid Gel Stain (10,000× Concentrate in DMSO)	ThermoFisher	S11494
40% Acrylamide/Bis Solution, 19:1	Biorad	1610144
TEMED	Merck	T7024-50ML
Ammonium persulfate	Merck	A3678-25G
RNA Gel Loading Dye (2×)	ThermoFisher	R0641
RiboRuler Low Range RNA Ladder, ready-to-use	ThermoFisher	SM1833
GeneRuler 50 bp DNA Ladder	ThermoFisher	SM0371
DMSO	Merck	D8416-250ML
dNTP Mix (10 mM each)	ThermoFisher	R0192

**Critical Commercial Assays**		

CloneJET PCR Cloning Kit	ThermoFisher	K1231
NovaBlue™ Competent Cells	Merck	69825
TRI Reagent™ Solution	ThermoFisher	AM9738
Ribo-Zero rRNA Removal Kit (Gram-Positive Bacteria)	Illumina	N/A
DNase I	Roche	10104159001
RNeasy MinElute Cleanup Kit	Eppendorf	74204
Oligo Clean & Concentrator Kit	Zymo Research	D4060
RNA Fragmentation Reagents	ThermoFisher	AM8740
Gel Breaker Tubes	IST Engineering	3388-100
Costar Spin-X columns	Merck	CLS8162
GlycoBlue Coprecipitant	ThermoFisher	AM9515
DNase I recombinant, RNase-free	Merck	4716728001
RNA 5′ Polyphosphatase	Epicentre	RP8092H
T4 RNA Ligase 1 (ssRNA Ligase)	NEB	M0204S
T4 polynucleotide kinase	ThermoFisher	EK0031
SUPERase-In RNase Inhibitor (20 U/μL)	ThermoFisher	AM2696
5′ DNA Adenylation Kit	NEB	E2610L
T4 RNA Ligase 2, Truncated	NEB	M0242L
RNAClean XP Kit	Beckman Coulter	A63987
AMPure XP, 60 mL	Beckman Coulter	A63881
TGIRT®-III reverse transcriptase	InGex	TGIRT50
Phusion High-Fidelity PCR Master Mix with HF Buffer	ThermoFisher	F531S
Agilent DNA 1000 kit	Agilent	5067-1504
CloneJET PCR Cloning Kit	ThermoFisher	K1231

**Experimental Models: Organisms/Strains**		

*Listeria monocytogenes* EGDe	ATCC	BAA-679

**Oligonucleotides**		

UCCCUACACGACGCUCUUCCGAUCU	5′RA (Ribooligonucleotide)	N/A
5phos/AGATCGGAAGAGCACACGT CTGAACTCCAG/3ddC	3′DA	N/A
CTGGAGTTCAGACGTGTGC TCTTCCGATCT	RT primer	N/A
AATGATACGGCGACCACCGAG ATCTACACTCTTTCCCTACACG ACGCTCTTC	LibAmp_F	N/A
CAAGCAGAAGACGGCATACGA GATCGTGATGTGACTGGAGTT CAGACGTGTGCT	LibAmp_RPI1_R	N/A
CAAGCAGAAGACGGCATACGA GATACATCGGTGACTGGAGTTC AGACGTGTGCT	LibAmp_RPI2_R	N/A
CAAGCAGAAGACGGCATACGA GATTGGTCAGTGACTGGAGTTC AGACGTGTGCT	LibAmp_RPI4_R	N/A
CAAGCAGAAGACGGCATACGA GATCACTGTGTGACTGGAGTTC AGACGTGTGCT	LibAmp_RPI5_R	N/A
CAAGCAGAAGACGGCATACGA GATATTGGCGTGACTGGAGTTC AGACGTGTGCT	LibAmp_RPI6_R	N/A
CAAGCAGAAGACGGCATACGAG ATGATCTGGTGACTGGAGTTCAG ACGTGTGCT	LibAmp_RPI7_R	N/A
CAAGCAGAAGACGGCATACGAG ATTACAAGGTGACTGGAGTTCAG ACGTGTGCT	LibAmp_RPI12_R	N/A
CAAGCAGAAGACGGCATACG AGATCGGGACGGGTGACTGG AGTTCAGACGTGTGCT	LibAmp_RPI16_R	N/A
AATGATACGGCGACCACCGAGATC	Enrich_F	N/A
CAAGCAGAAGACGGCATACGAGAT	Enrich_R	N/A

**Other**		

Shaking water bath	N/A	N/A
Incubator for 15 mL tubes	N/A	N/A
Cooling centrifuge for 15/50 mL tubes	N/A	N/A
Cooling centrifuge for 1.5/2 mL tubes	N/A	N/A
Nanodrop spectrophotometer	ThermoFisher	N/A
Mini-Beadbeater 8	Biospec	N/A
0.1 mm zirconium beads	Biospec	11079101z
Microlance 3 Needles 0.6 × 25 mm	BD	REF 300800
SpeedVac Concentrator	ThermoFisher	N/A
Magnetic stand for 1.5 mL tubes	N/A	N/A
Thermocycler with a heated lid	N/A	N/A
2100 Bioanalyzer Instrument	Agilent	G2939BA
Mini Protean system for gel electrophoresis including plates and combs	BioRad	N/A
Blue light transilluminator	N/A	N/A

**Software and Algorithms**		

Bowtie 2	(Langmead and Salzberg, 2012)	http://bowtie-bio.sourceforge.net/bowtie2
SAMtools	(Li et al., 2009)	http://www.htslib.org/doc/samtools.html
Integrative Genomics Viewer	(Thorvaldsdottir et al., 2013)	http://software.broadinstitute.org/software/igv/
HTseq framework	(Anders et al., 2015)	https://pypi.org/project/HTSeq/

## RESOURCE AVAILABILITY

### Lead Contact

Further information and requests for resources and reagents should be directed to and will be fulfilled by the Lead Contact, Jörgen Johansson (Jorgen.johansson@umu.se).

### Materials Availability

This study did not generate new unique reagents.

### Data and Code Availability

This study did not generate any unique datasets or code.

## MATERIALS AND EQUIPMENT

**Table:** Stocks of 6% and 15% Polyacrylamide Solutions with Urea

Reagent	6%	15%
Urea	48 g	48 g
40% AA:bisAA (19:1)	15 mL	37.5 mL
10 × TBE	10 mL	10 mL
RNase free water	To 100 mL	To 100 mL
**Total**	**100 mL**	**100 mL**

***Note:*** Dissolve all components at moderate heating and spinning. For example, use a 50°C water bath with occasional stirring or a heater and magnetic stirrer set to 70–80°C.

**Table:** BHI (BD) Buffered with MOPS

Reagent	Final Concentration	Volume (mL)
MOPS buffer, pH 7.3 (1 M)	50 mM	50
BHI broth	1 ×	950
**Total**		**1,000**

***Note:*** Prepare MOPS buffer from MOPS and adjust pH with NaOH.

**Table:** DMS Quenching Solution

Reagent	Final Concentration	Volume (mL)
Isoamyl alcohol	50%	100
β-mercaptoethanol	30%	60
RNase free water	20%	40
**Total**		**200**

**Table:** DMS Washing Solution

Reagent	Final Concentration	Volume (mL)
β-mercaptoethanol	30%	60
RNase free water	70%	140
**Total**		**200**

**Table:** Cell Disruption Solution

Reagent	Final Concentration	Quantity
Glucose	10%	10 g
Tris-HCl, pH 7.6 (1 M)	12.5 mM	1.25 mL
EDTA, pH 8.0 (0.5 M)	5 mM	1 mL
RNase free water		To 200 mL
**Total**		**200 mL**

**Table:** Dephosphorylation Buffer (5×)

Reagent	Final Concentration	Quantity, mL
MES buffer, pH 6.0 (1 M)	500 mM	5
MgCl_2_ (1 M)	50 mM	0.5
RNase free water		4.5
**Total**		**10**

***Note:*** MES buffer can be prepared from MES monohydrate powder and pH adjusted with NaOH or can be purchased already prepared.

**Table:** Reverse Transcription Buffer (5×)

Reagent	Final Concentration	Quantity, μL
Tris-HCl, pH 8.3 (1 M)	250 mM	250
KCl (1 M)	375 mM	375
MgCl_2_ (1 M)	15 mM	15
RNase free water		360
**Total**		**1,000**

**CRITICAL:** Acrylamide/Bis-acrylamide and β-mercaptoethanol pose a significant health risk. Refer to your country guidelines on working with these chemicals.

## STEP-BY-STEP METHOD DETAILS

### Growth of *L. monocytogenes* Bacteria – Days 1–3

**Timing: 30 min (day 1)**

***Note:*** The protocol was developed to determine the structure of 5′ UTRs in the human bacterial pathogen *Listeria monocytogenes*, but could in principle be applied to any other bacterial species.

1. Using an inoculation loop, plate *L. monocytogenes* cells on a Brain Heart Infusion (BHI) agar plate. Grow for 16 h (overnight) in a 37°C incubator.

**Timing: 30 min (day 2)**

2. The next day pick a colony and transfer it to 20 mL of BHI broth. Grow for 16 h (overnight) in a water incubator at 37°C and 150 rpm shaking.

**Timing: 3–5 h (day 3)**

3. The next day transfer 0.25 mL of the overnight culture to 25 mL of prewarmed BHI medium (dilution 1:100) and grow until the mid-logarithmic growth phase at 37°C and 150 rpm shaking.

***Note:*** Not only BHI, but also LB broth and chemically defined media (Whiteley et al., 2017) can be used to grow *L. monocytogenes*. Depending on the medium type and source, the genetic background of bacteria and equipment used to measure OD, the growth dynamics and OD values can vary considerably. Therefore, prior to the main experiment it is necessary to grow bacteria and plot the growth curves. Based on these data, optimal OD to perform dimethyl sulphate (DMS) treatment can be determined. Also, it is important to have the necessary volume of bacteria for DMS treatment to get sufficient RNA for further experiment. In this protocol we perform DMS treatment of *L. monocytogenes* in the mid-logarithmic growth phase (Ignatov et al, 2020).

### DMS Treatment - Day 3

**Timing: 1 h (day 3)**

4. Prewarm 15 mL tube to 37°C on the incubator. Using a prewarmed Pasteur pipette rapidly transfer 5 mL bacterial culture to the 15 mL tube.

**CRITICAL:** Do not allow the culture to cool down. Temperature is one of the main parameters affecting RNA structure. Furthermore, many bacteria possess very sensitive systems to detect temperature decrease and activate the cold shock response, which might affect the experiment results.

5. Using a 1 mL tip transfer 150 μL DMS to 5 mL of the culture (final DMS concentration 3%). Adjust the pipette sampling volume to 500 μL and using the same tip, pipette the culture up and down 10 times. Incubate the mixture for 3 min. Every 30 s pipette the mixture up and down.

**CRITICAL:** DMS and β-mercaptoethanol are highly toxic and a special protective equipment should be used when working with these chemicals. Therefore, refer to your country safety regulations when working with DMS and β-mercaptoethanol.

***Note:*** DMS does not dissolve in water solutions completely and without thorough mixing, DMS accumulates at the bottom of the tube.

6. Inactivate DMS by adding 6 mL Quenching solution and vigorously shaking the tube.
7. Collect bacteria by centrifugation at 5,000 × *g* for 10 min at +4°C. Wash the pellet with 5 mL ice cold 30% β-mercaptoethanol solution. Pulse centrifuge and completely discard the supernatant. Immediately proceed to RNA isolation [Problem 1].

### RNA Isolation – Days 3–4

**Timing: 2–3 h (day 3)**

***Note:****L. monocytogenes* is a Gram-positive bacterium with a thick cell wall. To isolate RNA the cell wall is disrupted by mechanical grinding followed by acid guanidinium thiocyanate-phenol-chloroform extraction. For other bacterial species the cell lysis protocols can be different.

8. Dissolve the bacterial pellet in 400 mL ice-cold Cell Disruption solution.
9. Transfer the cell suspension to the pre-cooled 2 mL screw-cap tube containing 500 μL of 0.1 mm zirconium beads (Biospec) and 500 μL of acid phenol (pH 4.5). Keep the tubes on ice.

**CRITICAL:** Phenol, TRI reagent and Chloroform are highly toxic and a special protective equipment should be used when working with these chemicals. Therefore, refer to your country safety regulations when working with Phenol, TRI reagent and Chloroform.

***Note:*** It is important to keep cells at low temperature during all preparations before addition of TRI reagent. This helps to minimize the potential activity of cellular RNases.

10. Disrupt the cells on a BeadBeater 8 (Biospec) cell disruptor at maximum speed for 30 s.

***Alternatives:*** other cell disruptors can be used instead of BeadBeater. As an example, we successfully tested Fastprep-24 (MP Biomedicals).

11. Centrifuge the disrupted cells at 12,000 × *g* for 5 min at +4°C. Collect the upper aqueous phase and transfer to a new 2 mL tube.
12. Add 1 mL of TRI reagent solution (ThermoFisher) and vigorously shake the tube by hands. Incubate the tube on the bench for 5 min.
13. Add 200 μl of Chloroform, vigorously shake the tube by hands and incubate on ice for 5 min.
14. Centrifuge the tube at 12,000 × *g* for 5 min at +4°C. Collect the upper aqueous phase and transfer to a new 2 mL tube. Add 500 μL of Chloroform and vigorously shake the tube by hands.
15. Centrifuge the tube at 12,000 × *g* for 5 min at +4°C. Collect the upper aqueous phase and transfer to a new 1.5 mL tube. Add isopropanol at 0.7× volume of the aqueous phase. Mix by vortexing and incubate at −20°C for 1 h or longer.

**Pause Point:** at this step the sample can be stored for up to one week at −20°C.

**Timing: 2 h (day 4)**

16. Precipitate RNA by centrifuging the tube at 20,000 × *g* for 30 min at +4°C. Carefully discard the supernatant and wash the pellet with 500 μL of 80% ethanol.
17. Centrifuge the tube at 20,000 × *g* for 5 min at +4°C and completely discard the supernatant. Dry the RNA pellet for 10 min on the bench and dissolve in 50 μl of RNase free water.
18. Measure the RNA concentration on nanodrop and check its quality on agarose gel [Problem 2][Problem 3].

**Pause Point:** The isolated DMS modified RNA can be stored at −20°C for a month or at −80°C for up to 6 months.

### RNA Treatment with DNase I, 5′ Polyphosphatase and Ribosomal RNA Depletion - Day 4

**Timing: 6–8 h (day 4)**

***Note:*** Ribosomal RNA constitutes up to 90% of the RNA content in bacterial cells. Therefore, to obtain a sufficient coverage of other transcripts it is necessary to remove it.

In our original work we used Ribo-Zero rRNA Removal Kit (Gram-Positive Bacteria) (Illumina). However, since then the kit has been discontinued by Illumina. As a substitute we suggest riboPOOL kit (siTOOLs Biotech); RiboMinus depletion kit (K155014, Thermofisher) or Ribo-Zero Plus rRNA depletion Kit (Illumina) but none of these kits have been tested by us.

***Note:*** To get enough RNA for subsequent steps of library preparation we started with 10 μg of DMS-treated total RNA.

19. Treat 10 μg of total RNA sample with DNase I (Roche) for 30 min at 37°C.
20. Purify RNA on RNeasy MinElute (Qiagene) columns according to the protocol from the manufacturer, elute from the column with 15 μL of RNase free water.
21. Remove γ and β phosphates from 5′-triphosphorylated RNAs by treatment with RNA 5′ polyphosphatase (Epicentre):

**Table:** 

Reagent	Amount
RNA sample	15 μL
RNA 5′ Polyphosphatase 10× Reaction Buffer	4 μL
RNase free water	19 μL
RNA 5′ Polyphosphatase (20 Units)	2 μL
**Total**	**40 μL**

22. Purify RNA on RNeasy MinElute columns according to the protocol from the manufacturer, elute with 15 μL of RNase free water.
23. Deplete ribosomal RNA using appropriate kit (see above). Adjust the volume of RNA solution to a final volume 20 μL with RNase free water.

**Pause Point:** The sample depleted of ribosomal RNA can be stored at −20°C for up to one week or at −80°C for up to one month.

### Ligation of the 5′ Adaptor, Fragmentation and Size Selection – Day 5

**Timing: 10 h (day 5)**

24. Concentrate the RNA sample on a SpeedVac Concentrator (ThermoFisher) to 4.5 μl.
25. Ligate the PAGE-purified 5′ RA:
a. Add 1 μL of 15 μM PAGE-purified 5′ RA and mix the reaction mixture by pipetting.
b. Heat for 3 min at 65°C in a thermocycler with hot lid and cool on ice.
c. Assemble the ligation reaction:

**Table:** 

Reagent	Final Concentration	Volume (μL)
T4 RNA Ligase Reaction Buffer	1×	2
RNA sample + 5′RA		5.5
DMSO	10%	2
SUPERase-In RNase Inhibitor		0.5
10 mM ATP	1 mM	2
T4 RNA Ligase 1	10 units	1
RNase free water		7
**Total**		**20**

d. Incubate for 2 h at room temperature (∼23°C).

***Note:*** From this step of the protocol we suggest introducing a negative control sample (K-): the 5′ RA ligation reaction set up without bacterial RNA. All subsequent steps should be the same for the samples and the negative control. After purification of Illumina cDNA library, the negative control should not have any products visible on electrophoresis. This guarantees that the cDNA libraries represent the sequences of bacterial RNAs and not any artefacts.

26. Ethanol precipitate RNA with ligated 5′RA
a. Add 70 μL of RNase free water, 10 μL of 3 M sodium acetate (pH 5.0), 2 μL of GlycoBlue coprecipitant and 300 μL of 100% ethanol. Vortex and incubate for 1 h at −20°C.
b. Precipitate RNA by centrifuging the tube at 20,000 × *g* for 30 min at +4°C. Carefully discard the supernatant and wash the pellet with 500 μL of 80% ethanol.
c. Centrifuge the tube at 20,000 × *g* for 5 min at +4°C and completely discard the supernatant. Dry the RNA pellet for 10 min on the bench and dissolve in 9 μL of RNase free water.

***Note:*** After depletion of ribosomal RNA, the quantity of RNA decreases significantly and for ethanol precipitation it is necessary to use a coprecipitant.

27. Perform RNA fragmentation with RNA fragmentation reagents (ThermoFisher):
a. Add 1 μL of the 10× Fragmentation Buffer, mix and spin briefly.
b. Incubate at 70°C for 3.5 min in a thermocycler with the heating lid.
c. Add 1 μL of the Stop Solution, mix, spin briefly and place the sample on ice.

***Note:*** The fragmentation parameters were empirically chosen to fragment *L. monocytogenes* RNAs to lengths of approximately 70–350 nucleotides (nts). If using another fragmentation method or bacterial species, it is advisable to calibrate the fragmentation conditions.

**Figure 1:**
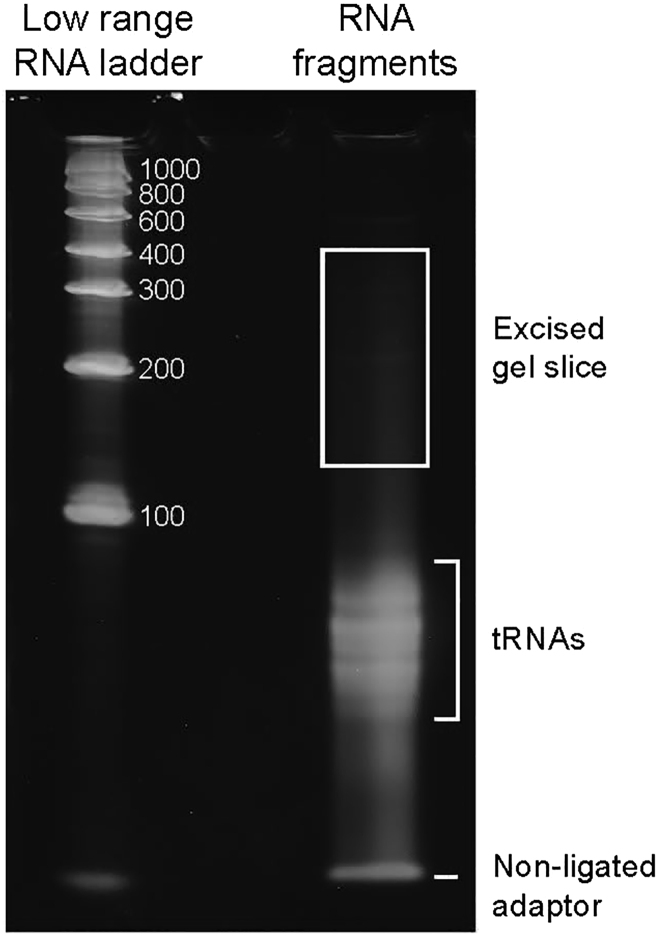
Size Selection of RNA Fragments The RiboRuler Low Range RNA ladder (ThermoFisher) is shown to the left. Numbers reflect sizes in nucleotides. The area of the excised piece of gel is within the square.

28. Resolve RNA on 6% denaturing polyacrylamide gel and isolate the fragments having lengths from 125 to 400 nts (Figure 1):

a. Pour 6% Urea-PAA gel using plates and the 5 well 1 mm wide combs of the Mini Protean system (Biorad):

**Table:** 

Reagent	Amount
6% Urea-PAA stock	9.91 mL
10% Ammonium persulfate	80 μL
TEMED	10 μL
**Total**	**10 mL**

b. Mix the fragmented RNA with 12 μL of 2× RNA loading dye (ThermoFisher), heat the mixture at 70°C for 10 min and chill on ice.
c. Apply the RNA sample to the gel in parallel with the RiboRuler Low Range RNA ladder (ThermoFisher) and run until the bromophenol blue dye migrates for 3 cm.

***Note:*** There is no need to extensively separate the RNA-fragments on the gel. Even after a short migration time, the non-ligated adapters and abundant tRNA species will be separated from the mRNA fragments. The short migration also decreases the size of the excised fragment, thereby decreasing the gel volume and increasing the recovery efficiency.

d. Stain the gel with SYBR gold and visualize on a blue light transilluminator. Excise the gel fragments with the lengths between 125 and 400 nucleotides.

***Note:*** the 6% PAA gel is very fragile and should be treated with care.

e. Disintegrate the gel fragments. With a hot needle punch ∼0.5 mm hole in the bottom of a 0.5 mL tube. Place the gel fragments in the 0.5 mL tube and insert it into a 2 mL tube. Centrifuge for 10 min at > 10 000 g.
f. Add 300 μL of 300 mM NaCl solution to the crushed gel and incubate for 2 h at 50°C and shaking at 300 rpm.
g. Transfer the gel slurry to the Costar Spin-X column and centrifuge for 3 min at 10,000 × *g* to remove the gel pieces.
h. Precipitate RNA by adding 2 μL GlycoBlue co-precipitant and 900 μL 100% EtOH, incubating at 20°C for 1 to 16 h and spinning at 20 000 g for 30 min at 4°C.
i. Resuspend the purified RNA fragments in 10 μL RNase-free water.

**Pause Point:** This RNA sample can be stored at −20°C for a week.

### Ligation of the 3′ Adaptor – Day 6

**Timing: 7 h (day 6)**

29. Dephosphorylate the 3′ ends of RNA fragments with T4 Polynucleotide Kinase.
a. Set up the following reaction:

**Table:** 

Reagent	Amount, μL
5× Dephosphorylation buffer	4
RNA sample	10
RNase free water	4.5
T4 Polynucleotide Kinase	1
SUPERase-In RNase Inhibitor	0.5
**Total**	**20**

b. Incubate at 37°C for 30 min.
30. Purify dephosphorylated RNA fragments with RNeasy MinElute Cleanup kit (Qiagen). Modify steps 1 and 2 of the protocol: to 20 μL of PNK reaction add 80 μL of RNase free water and 350 μL RLT buffer. Thoroughly mix the sample and add 550 μL of 100% ethanol. Proceed with the conventional protocol for RNeasy MinElute Cleanup kit.

***Note:*** the increased volume of ethanol added at the step 2 improves absorbance of RNA fragments shorter than 200 nts to the RNeasy MinElute column, preventing their loss during purification.

31. Concentrate RNA sample on a SpeedVac Concentrator to 7.5 μL.
32. Ligate adenylated 3′DA oligo using T4 RNA Ligase 2, Truncated (NEB).
a. Set up the following reaction:

**Table:** 

Reagent	Amount, μL
10× T4 RNA Ligase Reaction Buffer	2
RNA sample	7.5
adenylated 3′DA (15 μM)	1
50% PEG8000	8
SUPERase-In RNase Inhibitor	0.5
T4 Polynucleotide Kinase	1
**Total**	**20**

b. Incubate for 2 h at the room temperature (∼23°C).
33. Ethanol precipitate the reaction mixture:
c. Add 70 μL of RNase free water, 10 μL of 3 M sodium acetate (pH 5.0), 2 μL of GlycoBlue coprecipitant and 300 μL of 100% ethanol. Vortex and incubate for 1 h at −20°C.
d. Precipitate RNA by centrifuging the tube at 20,000 × *g* for 30 min at +4°C. Carefully discard the supernatant and wash the pellet with 500 μL of 80% ethanol.
e. Centrifuge the tube at 20,000 × *g* for 5 min at +4°C and completely discard the supernatant. Dry the RNA pellet for 10 min on the bench and dissolve in 20 μL of RNase free water.
34. Remove non-ligated 3′DA using RNAClean XP Kit. Follow the Protocol provided by the manufacturer but with the following modifications: 1) use the 1.6 × volume of magnetic beads to sample ratio (i.e. add 32 μL of beads to 20 μL of sample) 2) at the final step, elute the purified RNA from beads with 30 μL of RNase free water.

**Pause Point:** This RNA sample can be stored at −20°C for a week.

### Reverse Transcription Using TGIRTIII Enzyme – Day 7

**Timing: 5 h (day 7)**

35. Concentrate RNA solution on a SpeedVac Concentrator to 4.5 μL.
36. Perform reverse transcription with TGIRTIII enzyme (InGex)(Mohr et al., 2013, Zubradt et al., 2017):
a. Mix 4.5 μL of the RNA sample with 1 μL of 1 μM RT primer and 2 μL of 5× Reverse transcription buffer. Heat for 2 min at 80°C using the thermocycler with the heated lid. Transfer the tube to the room temperature (∼23°C) and allow RT primer to anneal for 5 min.
b. Add the other components to set up the final reaction:

**Table:** 

Reagent	Amount, μL
RNA sample, RT primer (1μM), Reverse transcription buffer (5×)	7.5
dNTPs mix (10 mM)	1
SUPERase Inhibitor	0.5
DTT (0.1 M)	0.5
TGIRT-III enzyme (200 u/μl)	0.5
**Total**	**10**

***Note:*** Use freshly prepared 0.1M dithiothreitol (DTT).

c. Incubate reaction at 57°C for 2 h in a thermocycler with a heated lid.
d. Add 1 μL 5 M NaOH, pipette up and down to mix, and incubate at 95°C for 3 min.
37. Ethanol precipitate the synthesized first strands of cDNA:
a. Add 160 μL of RNase free water, 20 μL of 3 M sodium acetate (pH 5.0), 2 μL of GlycoBlue co-precipitant and 600 μL of 100% ethanol. Vortex and incubate for 1 h at −20°C.
b. Precipitate the cDNA first strands by centrifuging the tube at 20,000 × *g* for 30 min at +4°C. Carefully discard the supernatant and wash the pellet with 500 μL of 80% ethanol.
c. Centrifuge the tube at 20,000 × *g* for 5 min at +4°C and completely discard the supernatant. Dry the pellet for 10 min on the bench and dissolve in 20 μL of nuclease free water.

**Pause Point:** The first strands of cDNA can be stored at −20°C for a week or at −80°C for longer period.

### Amplification of cDNA Libraries – Days 7–8

***Note:*** The amplification is performed by two rounds of PCR with several clean up steps to deplete the short DNA fragments, mostly represented by primers and adaptor dimers.

***Note:*** If you plan to sequence several DMS-MaPseq libraries with a single run on Illumina sequencing machine, carefully pick the LibAmp_RPIXX_R primers for amplification. The variable parts of these primers correspond to reverse complement of Illumina index sequences. To check the compatibility of different index adapters with each other, refer to Index Adapters Pooling Guide from Illumina.

**Timing: 4 h (day 7)**

38. Run the first round of PCR to amplify cDNA libraries.
a. Set up the PCR reaction:

**Table:** 

Reagent	Amount, μL
2× Phusion Master Mix	12.5
Primer LibAmp_F (10 μM)	1.25
Primer LibAmp_RPIXX_R (10 μM)	1.25
cDNA first strands	10
**Total**	**25**

b. Run the PCR reaction on a thermocycler with the heated lid:

**Table:** 

Temperature	Time	Cycles
98°C	30 s	1
98°C	10 s	12
64°C	20 s	
72°C	30 s	
72°C	5 min	1

39. Purify the PCR product using AMPure XP beads. Use 1:1 volume ratio of AMPure XP beads and PCR reaction (i.e. mix 25 μL of beads with 25 μL of PCR reaction). Elute from the beads with 40 μL of nuclease-free water.

***Note:*** the decreased ratio of beads to samples allows to deplete not only the PCR primers, but also very short cDNA fragments and adaptor dimers.

40. Run the second round of PCR to amplify cDNA libraries.
a. Set up the PCR reaction:

**Table:** 

Reagent	Amount, μL
2× Phusion Master Mix	25
Primer Enrich_F (10 μM)	2.5
Primer Enrich_R (10 μM)	2.5
cDNA first strands	20
**Total**	**50**

b. Run the PCR reaction on a thermocycler with the heated lid:

**Table:** 

Temperature	Time	Cycles
98°C	30 s	1
98°C	10 s	4–12
68°C	20 s	
72°C	30 s	
72°C	5 min	1

***Note:*** It is advisable to first calibrate the number of PCR cycles (Figure 2A). The product of the second PCR should be collected at the exponential phase of amplification to minimize amplification biases. Therefore, first perform a calibrating PCR by collecting 5 μL of PCR products from different cycles (e.g. 4, 6, 8, 10 and 12) and resolve them on 1.5% agarose gel along with GeneRuler 50 bp DNA ladder (ThermoFisher). Find the optimal cycles generating sufficient product for further work, but where the concentration has not yet reached saturation. In our experience, 4 to 6 cycles of the second PCR round is sufficient to get enough cDNA for Illumina sequencing.

**Timing: 3–5 h (day 8)**

**Figure 2:**
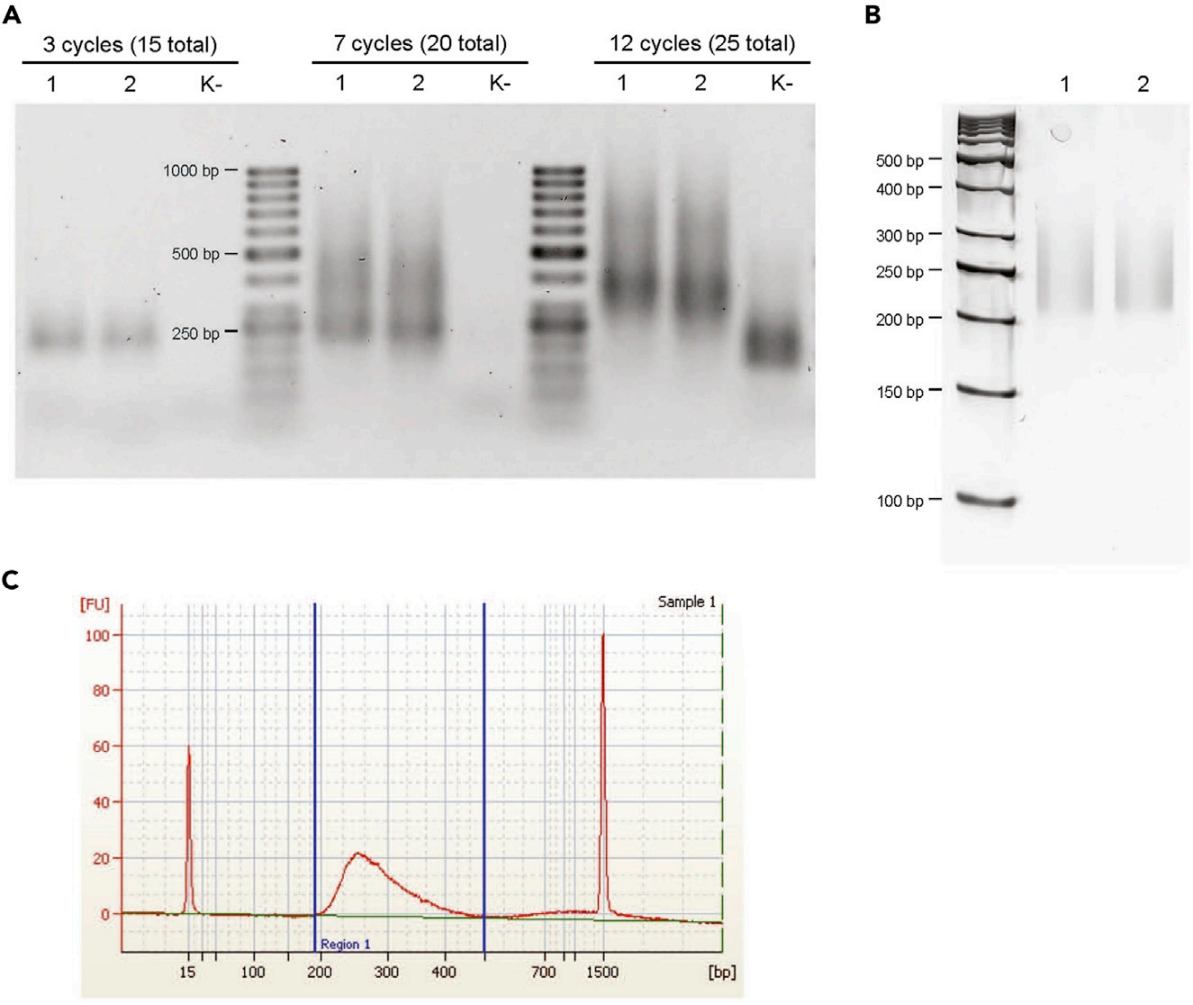
Amplification and Purification of Illumina cDNA Libraries (A) DNA fragments at different cycles of the second round of cDNA amplification. The fragments were resolved on agarose gel in parallel with GeneRuler 50 bp DNA Ladder (ThermoFisher). Samples 1 and 2 represent the biological replicates of the same library. Samples “K-“ is a negative control of the library prepared without the input of bacterial RNA. (B) The purified cDNA libraries 1 and 2 resolved on a native PAA gel along with GeneRuler 50 bp DNA Ladder. The libraries were amplified by 12 cycles of first round and 4 cycles of second round PCRs (16 cycles in total). (C) The purified cDNA library 1 resolved on an Agilent DNA 1000 chip.

41. Purify the PCR product using the RNeasy MinElute kit. Follow the protocol (i.e. do not add increased volume of ethanol to the mixture of PCR reaction and RLT buffer). At the final step elute from the column with 20 μL of nuclease-free water.
42. Purify the PCR product using AMPure XP beads. Use 1:1 volume ratio of AMPure XP beads and PCR reaction (i.e. mix 20 μL of beads with 20 μL of PCR product). Elute with 20 μL of nuclease-free water.
43. Measure the concentration of PCR product (library) on nanodrop. Resolve 5 μL of the library on 1.5% agarose or native PAA gel (Figure 2B) to study the distribution of the lengths of the fragments. For more precise determination of the lengths of the fragments use Agilent bioanalyzer with Agilent DNA 1000 Kit (Figure 2C).

***Note:*** At this step the negative control should not have any visible DNA sequences.

***Note:*** We recommend to clone the cDNA library (e.g. using the CloneJET PCR Cloning Kit from ThermoFisher) and sequence the inserts from 5 to 10 clones. This will ensure that both the 5′ and 3′ sequencing adapters are present in the fragments. Also, it will be possible to estimate the percentage of modified adenines and cytosines. Normally, 4%–6% of adenines and cytosines should be DMS modified. After mapping to the genome sequence, the modified bases can be identified as mismatches in cDNA sequences [Problem 4].

***Note:*** To obtain sequencing depth sufficient for comparison of 5′ UTR structures of highly expressed *L. monocytogenes* mRNAs we sequenced the libraries comprising 10 million reads. However, to compare the structures of moderately and low expressed transcripts it would be necessary to increase the sequencing volume to 50–100 million of reads per replicate.

## EXPECTED OUTCOMES

**Figure 3:**
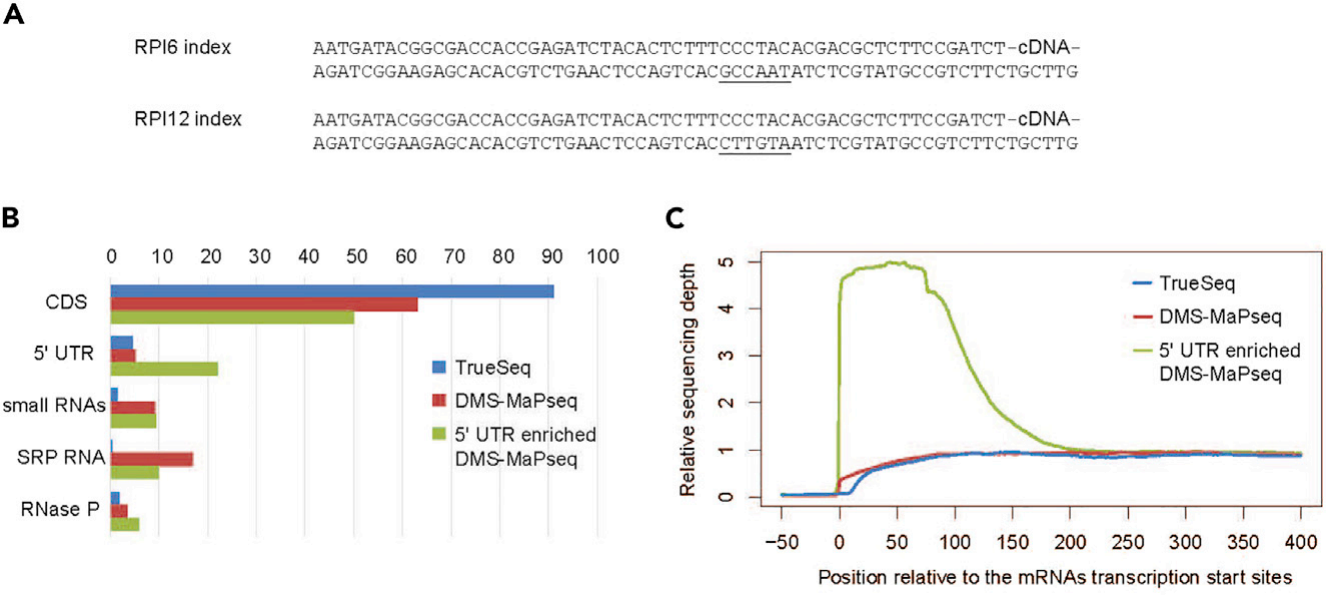
Characteristics of the Resulting 5′ UTR Enriched DMS-MaPseq Library (A) The sequences of cDNA adapters in the prepared library (after the second round of PCR amplification). (B) Representation of different RNA species generated by using TruSeq RNA Library Prep Kit, whole-transcriptome DMS-MaPseq and the 5′ UTR enriched DMS-MaPseq protocols, respectively. Further details of the library preparation are described in (Ignatov et al., 2020). (C) Sequencing coverage of *L. monocytogenes* mRNAs near the transcription start sites. The nucleotide positions of each *L. monocytogenes* mRNA and the region upstream of it were numbered relative to their transcription start sites. For each nucleotide of all mRNAs the RNA-seq coverage was calculated in three different libraries. The nucleotide coverages of each mRNA were divided by the mean coverage of the region 250 to 300 nt of this mRNA. Finally, the median coverage of each position was calculated for the three methods of library prediction preparation.

Accurate prediction of RNA secondary structures by DMS-MaPSeq approach (Zubradt et al., 2017) requires generation of cDNA libraries of good quality. This protocol presents a fast and reliable method to generate cDNA libraries with an increased sequencing depth for bacterial 5′ UTRs. Such libraries can be used for sequencing and subsequent structural determination and are identical to the libraries generated with TruSeq RNA Library Prep Kit v2 (Truseq single indexes – Illumina, Figure 3A). The generated libraries can be sequenced on Illumina machines using the same mode as for TruSeq libraries.

The depletion of ribosomal RNAs decreases their representation in the library to less than 25% and the size selection step (transcripts shorter than 100 nt are removed) depletes tRNAs. The majority of reads thus represent mRNAs and small RNAs, including highly abundant RNase P and 4.5S RNA (Figure 3B). Since the 5′ adaptors are ligated to RNA fragments having the γ and β phosphates removed prior to RNA fragmentation, the 5′ ends of bacterial mRNAs have increased sequencing coverage (Figure 3C).

## QUANTIFICATION AND STATISTICAL ANALYSIS

### Guideline for the Analysis of 5′ UTR Targeted DMS-MaPseq Data

***Note:*** the base pairing status of an adenine or cytosine nucleotide can be calculated as a ratio between the number of mismatches of cDNA sequence at that nucleotide and its transcriptional coverage. For DMS-MaPseq this ratiometric measure of base pairing does not require background correction (Zubradt et al., 2017). However, the parallel sequencing of DMS untreated sample can be useful to identify endogenous mRNA modifications (Zubradt et al., 2017).

***Note:*** The analysis of DMS-MaPseq data requires writing custom scripts in Python programming language. For that we suggest using the HTseq framework that contains multiple tools for processing of RNA-seq data (Anders et al., 2015).

**Figure 4:**
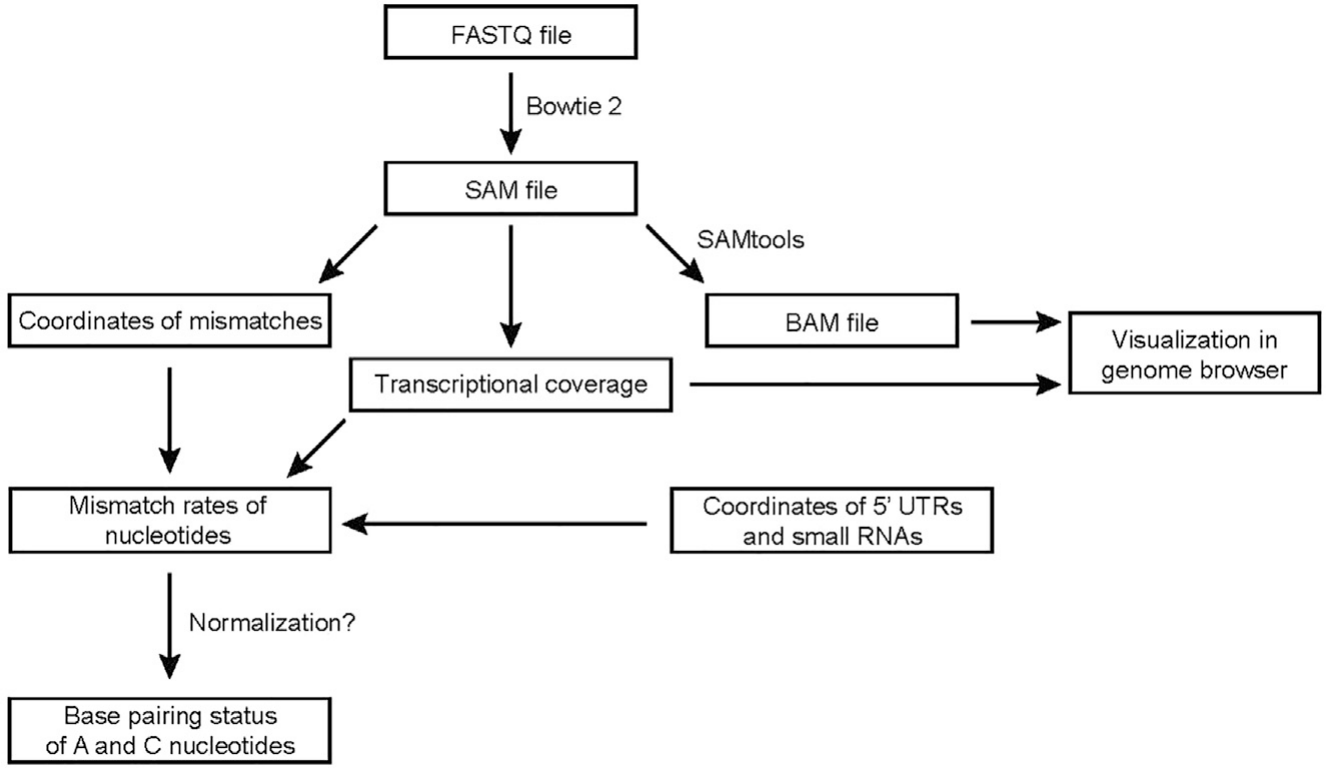
The Outline for Analysis of the 5′UTR Targeted DMS-MaPseq Data See text for further details.

1. Map the Illumina reads to *Listeria monocytogenes* EGD-e genome (NC_003210) with Bowtie 2 aligner (Langmead and Salzberg, 2012) using the --end-to-end --very-sensitive mode. This results in generation of SAM files recording the coordinates of aligned reads and mismatches encoded in cDNAs (Figure 4).

2. Using the SAMtools software (Li et al., 2009) convert SAM files to BAM files. Visualize BAM files with a genome browser, for example Integrative Genomics Viewer (Thorvaldsdottir et al., 2013). An alternative way to visualize transcriptional profile is to calculate the transcriptional coverage for each nucleotide programmatically and visualize the generated profile.
3. For further analysis the coordinates of the 5′ ends of mRNAs are necessary. For some species the coordinates of transcriptional start sites (TSS) can be obtained from literature: for *L. monocytogenes* EGDe the TSS coordinates were retrieved from (Wurtzel et al., 2012). In principle, the coordinates of TSSs can be determined using the data from the 5′UTR targeted DMS-MaPseq experiment itself. This can be done programmatically by searching for the steep increase of transcriptional coverage upstream of each start codon. Afterwards the identified TSSs should be manually checked using the transcriptional profile visualized in the genome browser. Please note however that our approach does not discriminate between the primary TSSs and the processed 5′ ends generated by the action of cellular RNases. To obtain the primary TSSs we suggest using the differential RNA-seq approach (Borries et al., 2011).
4. Define the mRNA regions for further analysis. In our case the genome regions corresponding to non-coding RNAs, 5′ UTRs and the first 30 nucleotides of coding sequences were selected for further analysis.
5. Use the HTseq framework to extract the coordinates of mismatches from the CIGAR string of the SAM files.
6. For each adenine and cytosine nucleotide of the selected regions, calculate the coverage as the number of reads mapped at that position and the number of mismatches in the mapped cDNAs.
7. For each nucleotide calculate the rate of mismatches by dividing the number of mismatches by the transcriptional coverage.

***Note:*** This ratiometric measure of base-pairing status depends on the degree of DMS modification of RNA. Therefore, the conditions of DMS treatment should be very reproducible. However, in some cases, for example when DMS treatment is performed at different temperatures, this is not achievable. In this case, we suggest normalization of the data. For that the average rate of mismatches in a sample should be calculated. The division of a nucleotide mismatch rate by the average rate in the sample will generate the normalized measure of base pairing status that we termed “DMS values” (Ignatov et al., 2020).

***Note:*** The obtained data on base-pairing status of adenines and cytosines can further be used to model RNA secondary structure and perform comparison of RNA structures in different samples. These further steps can be variable and we consider their description to be beyond the topic of our protocol. As a primer for further analysis we refer the reader to the review by Choudhary et al. (Choudhary et al., 2017).

## LIMITATIONS

The protocol has been designed to increase the sequence coverage of 5′UTRs. This decreases the proportion of reads from other parts of the transcript. If planning to obtain structural information of whole transcripts, rather use a whole-transcriptome DMS-MaPseq protocol.

***Note:*** It is possible to use Cap-Clip Acid Pyrophosphatase (Cambio #C-CC15011H) to hydrolyze the pyrophosphate bonds of the 5′-terminal m7GpppG "cap" of eukaryotic messenger RNAs. This decapping method allows to adapt our protocol for eukaryotic cells. However, the protocol was developed for bacteria and has not been comprehensively tested in eukaryotes.

## TROUBLESHOOTING

### Problem 1

The bacterial pellet is lost after DMS quenching.

### Potential Solution

The *Listeria* pellet is rather loose after DMS quenching and this also might be observed if using other bacterial species. It is therefore critical to run a pilot experiment and if necessary, increase the volume of bacterial culture to facilitate the precipitation of bacteria.

### Problem 2

Low RNA yield.

### Potential Solution

The library preparation requires at least 10 μg of high quality DMS-treated RNA. We obtained this quantity from 5 mL of mid-exponential *L. monocytogenes* culture. However, for other bacteria or growth conditions it may be necessary to increase the volume of the culture for DMS treatment. Another reason for low yield might be the inefficient cell lysis. When cells are disrupted on a Bead beater we noticed that the geometry of tubes for disruption can be important. Therefore, never use 1.5 mL screw-capped tubes, but only the 2 mL screw-capped tubes.

### Problem 3

Low RNA quality on agarose gel.

### Potential Solution

There can be several reasons for the compromised RNA quality. When DMS decomposes in water, sulfuric acid is generated and in the absence of a proper buffer, the pH can drop significantly. In this protocol, we used broth buffered with 50 mM MOPS pH 7.3. However, depending on DMS concentration and the time of treatment, it might be necessary to increase MOPS concentration to 100 mM. Another reason for RNA degradation might be the activity of bacterial or external RNases. Therefore, it is critical to use RNase free reagents and labware and always keep the samples on ice.

### Problem 4

Low amount of modified bases.

### Potential Solution

The fraction of modified adenines and cytosines should generally lie between 4% and 6%.

DMS activity depends on temperature, time of incubation and mixing regime. Therefore, for a new experimental setup it is advisable to first perform a series of pilot experiments with different incubation times and/or DMS concentrations in the range of 2%–5%.
